# A Peer Intervention Facilitates Trust and Improves Psychosocial Well-Being in Diverse, Low-Income Older Adults

**DOI:** 10.1093/geroni/igab046.021

**Published:** 2021-12-17

**Authors:** Ashwin Kotwal, Shannon Fuller, Janet Myers, Daniel Hill, Soe Han Tha, Alexander Smith, Carla Perissinotto

**Affiliations:** 1 University of California San Francisco, University of California San Francisco, California, United States; 2 Curry Senior Center, San Francisco, California, United States

## Abstract

We evaluate a peer outreach intervention to improve the psychosocial well-being of diverse, low-income older adults. Participants (N=74, Age 58-96 years) were recruited from an urban senior center and matched with peers who were >55 years old, received mental health training, and connected participants with health or social activities. We conducted surveys at baseline and 6-month follow-up for 2 years with validated measures of loneliness, social interaction, barriers to socializing, and depression, and thematically analyzed qualitative, semi-structured interviews conducted among a subset of participants (n=15) and peers (n=6). Participants were 58% male, 18% African-American, 19% Latinx, and 8% Asian. Over 2 years, participants experienced sustained reductions in loneliness (p=0.015), depression (p<0.001), and barriers to socializing (p<0.001). Qualitative interviews detailed the role of longitudinal relationships, program flexibility, and the matching process in facilitating trust, motivation, and improved mood. Results can inform larger efficacy studies and implementation of peer-driven community programs.

